# Lessons from history: mass mobilization and public health campaigns in China’s anti-schistosomiasis efforts (1950s–1980s)

**DOI:** 10.3389/fpubh.2026.1790023

**Published:** 2026-03-30

**Authors:** Qiu-Lan Guo, Hong-Bo Chen

**Affiliations:** 1 School of History, Culture and Tourism, Gannan Normal University, Ganzhou, China; 2 Third Clinical Medical College of Gannan Medical University, Ganzhou, China

**Keywords:** China, disease elimination, health promotion, mass mobilization, Patriotic Health Campaign, public health history, schistosomiasis control

## Abstract

**Background:**

Schistosomiasis is a neglected tropical disease (NTD) that posed a major public health challenge in China, with approximately 11.6 million people infected in 1949. This scoping review systematically examines the mass mobilization practices of China’s anti-schistosomiasis campaign during the 1950s–1960s and traces epidemiological outcomes through the early 1980s.

**Methods:**

Following the Arksey and O’Malley framework and PRISMA-ScR guidelines, data were collected from government documents, academic databases (PubMed, Web of Science, Scopus, CNKI), and historical archives. Narrative synthesis and SWOT analysis were employed to evaluate the mass mobilization model.

**Results:**

Despite 80% rural illiteracy and severe resource constraints, China mobilized millions of peasants through high-level political commitment, multi-sectoral coordination, integration of disease control with agricultural production, and culturally adapted health communication. These efforts contributed to reducing infected cases from 11.6 million to below 1 million by approximately 1982–1983 (>90% decline). However, SWOT analysis revealed inherent limitations: weak grassroots technical capacity, unsustainable campaign-style mechanisms, and formalism that compromised surveillance accuracy, leading to disease resurgence in some areas.

**Conclusion:**

Political mobilization and community participation can partially compensate for limited medical resources, but effective interventions require balancing mass participation with scientific professionalism and establishing institutionalized mechanisms. These historical lessons remain relevant for contemporary NTD elimination, “One Health” practices, and public health emergency responses, though application requires consideration of contextual differences across countries.

## 1 Introduction

Schistosomiasis, a neglected tropical disease, is caused by parasitic flatworms of the genus Schistosoma. It is estimated that globally, there are some 250 million infections with schistosomiasis and a further estimated 800 million people at risk of contracting the disease (1). China has played a very prominent role in the endemic areas for schistosomiasis in particular and has enjoyed an uninterrupted history of schistosome transmission for more than two millennia. The earliest recorded history in China dates to the Western Han Dynasty (206 BCE-8 CE), when inhabitants of the Yangtze River region contracted infections; in fact, the eggs of schistosomes have been found in an excavated interment of a noble lady from the Mawangdui tomb in China in excavations in the 1970s (2). At the establishment of the People’s Republic of China in 1949, schistosomiasis was an endemic disease in 12 provinces (municipalities and autonomous regions), all of which lie on and south of the Yangtze River; it was estimated to have infected 11.6 million people, with another zoonotic infection of 1.2 million cattle, while more than 100 million people were at risk (3). The disease in its very highly endemic areas caused ascites and hepatosplenomegaly, and chronic illness-related mortality was not uncommon; in consequence, “distressed villages” or “empty villages” and “widows’ villages” emerged because of schistosomiasis; as a result, schistosomiasis emerged as a serious threat to public health in rural areas and a potential limiting factor in agricultural production (4). Notably, while schistosomiasis was also endemic in countries such as Brazil, the Philippines, and Egypt, China’s approach was distinctive in its reliance on mass political mobilization and community-based snail elimination, in contrast to Brazil’s emphasis on chemotherapy-centered strategies (5), the Philippines’ more limited community engagement due to geographic fragmentation (6), and Egypt’s large-scale drug administration campaigns targeting *S. haematobium* and *S. mansoni* (7). These differences highlight the unique political and organizational dimensions of the Chinese model.

However, with a heavy disease burden, the public health system in the pre-existing China remained underdeveloped. Rural illiteracy rates in 1950s China reached approximately 80%, and peasants in general were not aware of the methods for the prevention and spread of schistosomiasis, and in some cases, the disease was thought to be caused by superstitions, such as the “water spirits” (8). At the same time, medical facilities were extremely underdeveloped, and staff in the medical sector were mainly found in major urban areas and were absent in a significant portion of rural China. Under such a situation, the Chinese government incorporated the control of schistosomiasis into the Patriotic Health Campaign and, for the first time, used a model for mobilizing a massive number of people to promote control of the disease at a huge scale. In 1955, Chairman Mao Zedong issued the directive ‘Schistosomiasis must be eliminated,’ thereby elevating it to national priority. Subsequently, the ‘National Program for Agricultural Development (1956–1967)’ was released in 1956 whose goal included the complete eradication of schistosomiasis in a period of no more than 7 years and associated with agricultural development and water conservancy construction (9). In 1958, in a declaration in Jiangxi Province, the county of Yujiang declared the eradication of schistosomiasis and became the first county in the whole country to do so. Later, Chairman Mao Zedong composed a poem titled “Farewell to the God of Plague” because of the event and had a clear significant demonstration effect and also increased political mobilization (10).

This large-scale control campaign achieved remarkable epidemiological outcomes. Through the mass snail elimination and treatment campaigns of the 1950s-1960s, the number of infected people declined from 11.6 million to below 1 million by approximately 1982–1983, a reduction of over 90%; the snail-infested area decreased from 14.3 billion square meters to approximately 3.5 billion square meters (3,500 km^2^), a reduction of over 75% (11). These figures should be interpreted with caution given the reporting limitations of the era, as discussed in Section 3.2. This achievement was remarkable in the international parasitic disease control field at the time and laid the foundation for China’s eventual elimination of schistosomiasis as a public health problem in 2015 (12), though recent analyses highlight ongoing challenges in the new era of schistosomiasis control and elimination (13). The WHO 2021–2030 Roadmap for Neglected Tropical Diseases explicitly identifies political commitment and community participation as key elements for achieving elimination targets, and China’s historical experience in schistosomiasis control provides a replicable mobilization model for resource-limited countries (14).

Existing literature on China’s anti-schistosomiasis campaign of the 1950s-1960s has primarily focused on three dimensions. Epidemiological studies have documented time-series data on infection rates, snail distribution, and control outcomes in detail, providing quantitative evidence for evaluating intervention effectiveness (15, 16). Political and medical history studies have analyzed the campaign’s relationship with nation-building, the Patriotic Health Campaign, and Maoist-era political mobilization from a macro perspective, revealing the political significance attributed to disease control (8, 17). Zhou (18) analyzed the evolution of health education and propaganda strategies in schistosomiasis control from the Mao era to the present from a cultural policy perspective. Ding (19) conducted a critical re-examination of the 1950s campaign, highlighting the sustainability limitations of the campaign-style approach. However, these studies have either focused on quantitative descriptions of epidemiological outcomes or emphasized macro-level narratives of political mobilization, lacking systematic analysis of the specific mechanisms of mass mobilization, implementation strategies, and operational-level lessons learned. How was mass mobilization implemented at the grassroots level? How did health education through different communication channels adapt to populations with high illiteracy rates? How was snail elimination integrated with agricultural production? What challenges did mass treatment face and what were the response strategies? These questions have not been adequately addressed in the existing literature. For example, existing studies have not systematically examined how the integration of snail elimination with agricultural production practically reconciled potential conflicts between disease control goals and farming productivity at the grassroots level, nor have they analyzed the specific mechanisms through which health education achieved behavioral change among populations with extremely limited literacy.

This review aims to fill this gap by employing a scoping review methodology to systematically examine the development of China’s anti-schistosomiasis campaign in the 1950s-1960s, identifying key milestones and policy evolution, and conducting in-depth analysis of the specific practical strategies of mass mobilization—including organizational integration with the Patriotic Health Campaign, health education and propaganda activities, community participation in snail elimination, and the organization and implementation of mass treatment. On this basis, this study applies the SWOT analysis framework to systematically evaluate the strengths and limitations of the mass mobilization model from both internal factors and external environment dimensions, identifying successful experiences and historical lessons. The significance of this review lies not only in providing a new analytical perspective for understanding China’s public health history but also in offering historical lessons for contemporary global public health practice—particularly NTD elimination programs, “One Health” practice, and public health emergency response. Given that over 1.6 billion people globally still require NTD interventions (1), summarizing and reflecting on the experiences and limitations of the mass mobilization model in 1950s-1960s China holds important practical significance for exploring large-scale disease control pathways under resource-limited conditions.

Accordingly, this review concentrates on the mass mobilization practices implemented during the 1950s-1960s, while examining their long-term epidemiological impact through approximately 1982–1983, when infected cases declined to below one million.

## 2 Methods

### 2.1 Study design

A scoping review methodology was employed to systematically examine the policy evolution, organizational implementation, and mass mobilization practices of China’s anti-schistosomiasis campaign in the 1950s–1960s. Scoping reviews are appropriate for exploratory research topics, particularly those involving diverse evidence sources including historical literature and policy documents (20). This review followed the scoping review framework proposed by Arksey and O’Malley, drawing on the basic principles of the PRISMA-ScR reporting guidelines (21). As this study focused on historical narrative synthesis rather than systematic evidence evaluation, formal quality assessment of literature was not performed. The study design comprised five stages: identifying research questions, searching relevant literature, screening literature, charting data, and collating, summarizing, and reporting results. This review focused on three core questions: What were the key milestones and development trajectory of China’s anti-schistosomiasis campaign in the 1950s-1960s? What were the specific practical strategies of mass mobilization mechanisms? What lessons does this historical experience offer for contemporary global public health? As this study involved historical policy analysis rather than human subjects research, ethical review approval was not required according to relevant regulations.

### 2.2 Data sources

Data sources for this review encompassed four categories: government official documents, academic databases, historical archives, and review literature. For government documents, policy documents, work reports, and statistical bulletins related to schistosomiasis control published on the official websites of the National Health Commission of the People’s Republic of China (formerly Ministry of Health) and the State Council were searched, with particular focus on foundational policy documents such as the 1955 “CPC Central Committee Directive on Eliminating Schistosomiasis” and the 1956 “National Program for Agricultural Development (1956-1967).” For academic literature, English-language publications were searched in PubMed, Web of Science, and Scopus databases using search terms including combinations of “schistosomiasis,” “China,” “control,” “elimination,” “mass mobilization,” and “Patriotic Health Campaign”; Chinese-language publications were searched in CNKI, Wanfang Data, and VIP databases using search terms including “blood fluke disease,” “control,” “mass movement,” and “Patriotic Health Campaign.” Literature searches were conducted between January and June 2024. Inclusion criteria were: (1) studies addressing schistosomiasis control policies, mass mobilization, or epidemiological outcomes in China; (2) government documents and archival materials related to the anti-schistosomiasis campaign; (3) publications in English or Chinese. Exclusion criteria were: (1) studies focusing exclusively on laboratory or clinical aspects without relevance to public health campaigns; (2) editorials or opinion pieces without substantive empirical or historical content. No time restrictions on publication dates were applied to comprehensively capture historical research literature, with priority given to systematic reviews and historical analyses published in the past decade. Initial screening was conducted by title and abstract review, followed by full-text assessment for relevance to the research questions. For historical archives, relevant reports from the People’s Daily database (1949–1970), the U.S. National Library of Medicine’s Chinese Public Health Posters Digital Collection, and materials from the Second Historical Archives of China were searched. Additionally, historical reviews of China’s schistosomiasis control published in journals such as Advances in Parasitology, Infectious Diseases of Poverty, and Acta Tropica, as well as relevant academic monographs, served as key references.

### 2.3 Data extraction and synthesis

Data extraction employed a pre-designed standardized form. Extracted content included: release dates, issuing authorities, core content, and policy objectives of key policy documents; time-series data on epidemiological indicators such as number of infected people, infection rates, and snail-infested areas; hierarchical structure and functional division of the organizational leadership system; specific strategies and implementation methods of mass mobilization; and control outcomes and historical evaluations.

Data synthesis employed narrative synthesis methods, organizing and analyzing extracted information by theme (22). The analysis began with descriptive examination of included literature to outline the timeline and key nodes of the anti-schistosomiasis campaign; this was followed by thematic categorization of different practical strategies of mass mobilization; the SWOT analysis framework (Strengths-Weaknesses-Opportunities-Threats) was then applied to systematically evaluate the internal strengths and weaknesses, external opportunities and threats of mass mobilization mechanisms (23). The SWOT analysis framework is widely applied in public health program evaluation and enables comprehensive understanding of the characteristics and limitations of interventions from both internal factors and external environment dimensions (24).

To ensure research quality, literature screening and data extraction were conducted by the research team. The accuracy of historical data was verified through cross-validation of multiple sources, with discrepancies between different sources noted where applicable. Specifically, cross-validation involved triangulating epidemiological figures reported in government documents against those cited in peer-reviewed publications and WHO reports; where discrepancies exceeded 10%, the primary source was identified, and the range of estimates was reported. For qualitative historical claims, corroboration from at least two independent sources was sought before inclusion in the synthesis.

The literature search identified a total of approximately 320 potentially relevant records across all databases and archival sources. After removal of duplicates (n ≈ 85), 235 records were screened by title and abstract, of which 128 were excluded as irrelevant. Full-text assessment was conducted for the remaining 107 records, resulting in 47 sources included in the final synthesis: 12 government policy documents and official reports, 28 peer-reviewed academic publications (including epidemiological studies, historical analyses, and systematic reviews), and 7 historical archival materials and digital collections. Given the heterogeneous nature of sources in this historical scoping review, a formal PRISMA flow diagram was not generated; however, the screening process followed the sequential stages outlined above.

## 3 The development and progress of anti-schistosomiasis campaign in China

### 3.1 Key milestones in the anti-schistosomiasis campaign

Schistosomiasis has a transmission history of over 2,100 years in China, with archaeological evidence indicating that Yangtze River basin residents were affected as early as the Western Han Dynasty (206 BCE–8 CE) (2). At the founding of the People’s Republic of China in 1949, schistosomiasis was endemic in 12 provinces (municipalities and autonomous regions) along and south of the Yangtze River, with an estimated 11.6 million infected people, 1.2 million infected cattle, and over 100 million people at risk (3). In heavily endemic areas, tragic “empty villages” and “widows’ villages” emerged, making schistosomiasis a major public health problem threatening rural population health and constraining agricultural production.

Table 1 summarizes the key milestones of the anti-schistosomiasis campaign from 1949 to 1968. Among these, Chairman Mao’s 1955 directive “Schistosomiasis must be eliminated” and Yujiang County’s 1958 declaration of schistosomiasis elimination were two landmark events—the former elevated schistosomiasis control to national strategic importance, while the latter generated a powerful demonstration effect (15). However, the Great Leap Forward movement of 1958–1960 and the subsequent 3 years of difficulty severely disrupted control work, and the campaign entered a low ebb following the outbreak of the Cultural Revolution in 1966.

**Table 1:** Key milestones of the anti-schistosomiasis campaign in China (1949–1968).

Year	Key milestones
1949	Founding of the People’s Republic of China; initial assessment identified 12 endemic provinces with an estimated 11.6 million infected people and 1.2 million infected cattle
1952	Launch of the Patriotic Health Campaign in response to alleged biological warfare during Korean War
1955	Chairman Mao issued the directive “Schistosomiasis must be eliminated”; establishment of the National Schistosomiasis Control Leading Group
1956	Release of “National Program for Agricultural Development (1956–1967)” proposing elimination of schistosomiasis within 7 years; integration with agricultural production and water conservancy
1957	Establishment of specialized anti-schistosomiasis stations at provincial and county levels; promotion of three-day antimony potassium tartrate (APT) treatment
1958	Yujiang County, Jiangxi Province declared elimination of schistosomiasis; Chairman Mao published the poem “Farewell to the God of Plague”
1963	Adjustment of control strategies following post-Great Leap Forward reassessment
1966–1968	Campaign activities disrupted during early Cultural Revolution period

Data synthesized from Li et al. (25), Zhang et al. (3), and historical policy documents.

### 3.2 The epidemiological profile before and during the campaign

**Figure 1:**
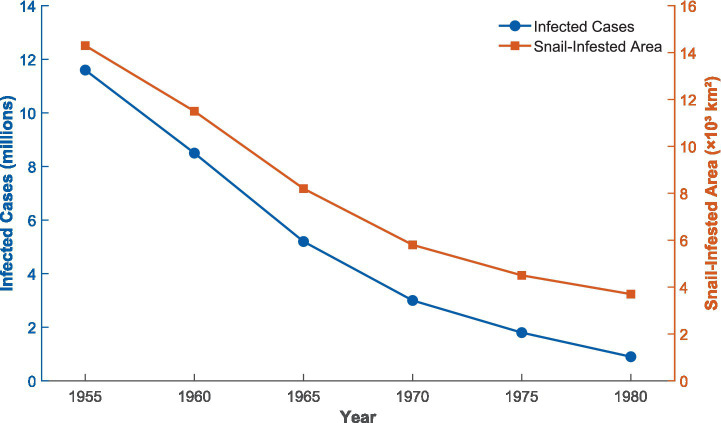
The estimated number of schistosomiasis cases (millions) and snail-infested areas (billion m^2^) from the 1950s to 1980s in China. Data sources: Cao et al. (11), Zhang et al. (16); WHO reports. Data shown from 1955 when systematic control campaigns began; 1949 baseline estimates were 11.6 million infected. The estimate of below 1 million infected by approximately 1982–1983 is based on national survey data reported in Cao et al. (11) and corroborated by Zhang et al. (16). Y-axis units: left axis = estimated number of cases (millions); right axis = snail-infested area (billion m^2^).

The epidemiological outcomes of the 1950s-1960s anti-schistosomiasis campaign can be evaluated through two core indicators: number of infected people and snail-infested areas (Figure 1). Before the campaign, the national number of schistosomiasis cases was approximately 11.6 million, with snail-infested areas of approximately 14.3 billion square meters (14,300 km^2^). Through large-scale control efforts, the number of infected people declined to below 1 million by approximately 1982–1983, a reduction of over 90%; snail-infested areas decreased to approximately 3.5 billion square meters (3,500 km^2^), a reduction of over 75% (11). Note: Exact figures vary slightly across sources due to differences in survey methodologies and reporting periods; the values cited here represent synthesized estimates from Cao et al. (11) and Zhang et al. (16), and are consistent with the trends depicted in Figure 1. This achievement was remarkable in the international parasitic disease control field at the time. By endemic type, China’s schistosomiasis endemic areas are classified into three categories: lake/marshland, hilly, and waterway types. Hilly and waterway endemic areas achieved relatively good control outcomes in the 1950s-1960s, with some counties and cities reaching transmission control or even elimination standards; however, lake/marshland endemic areas, primarily distributed in the Dongting Lake and Poyang Lake regions of the middle and lower Yangtze River, faced extremely challenging control due to complex ecological environments and vast snail-breeding areas, with relatively limited outcomes. These areas remain the focus and difficulty of China’s schistosomiasis control to this day (9, 16, 25, 26).

However, interpretation of epidemiological data from this period requires caution. During the Great Leap Forward period of 1958–1960, some areas experienced problems of exaggerated reporting of achievements, leading to distortions in published infection rate decline data (15). In 1963, the central government conducted a comprehensive review of schistosomiasis control work, discovering that some areas declared to have “eliminated” the disease experienced resurgence, prompting strategy adjustments emphasizing consolidation of achievements rather than blind pursuit of elimination targets. Notably, although schistosomiasis control institutions at all levels were severely disrupted in the early Cultural Revolution period after 1966, grassroots snail elimination and treatment work was not completely interrupted. In the early 1970s, as the political situation became relatively stable, control work gradually resumed in some areas. Particularly, the successful development and widespread application of praziquantel in the late 1970s fundamentally transformed the treatment landscape, providing technical support for the substantial decline in infection rates in the early 1980s (27). Therefore, the epidemiological outcomes of the early 1980s were the combined result of the foundation laid by mass mobilization in the 1950s-1960s and the technological advances of the late 1970s.

### 3.3 Organizational structure and leadership system

The anti-schistosomiasis campaign, particularly during its intensive phase in the 1950s-1960s, established a five-level organizational leadership system from central to grassroots levels, with core characteristics of political authority leadership, administrative mobilization implementation, and professional institutional support. At the central level, the National Schistosomiasis Control Leading Group was established in 1955, headed by a State Council Vice Premier, with members including officials from the Ministry of Health, Ministry of Agriculture, Ministry of Water Resources, and other relevant ministries and commissions, responsible for formulating national control policies, coordinating cross-departmental and cross-regional work, and allocating major resources (28). At the provincial level, Provincial Schistosomiasis Control Leading Groups were established, headed by key provincial leaders to coordinate provincial work, while Provincial Schistosomiasis Research Institutes or Control Stations were established as professional technical support institutions (2). At the county level, Schistosomiasis Control Command Headquarters were established, headed by county magistrates to integrate the forces of health, agriculture, water conservancy, and other departments, with county-level control stations serving as the main technical implementing institutions responsible for epidemic surveillance, snail surveys, and case diagnosis and treatment (16). At the township level, control stations or health center control groups served as the hub connecting county-level professional institutions with village-level mass organizations. Village-level control teams were the most grassroots implementing units, composed of village cadres, health workers, and activists, responsible for organizing community participation in snail elimination, assisting mobile medical teams in conducting surveys and mass treatment, and promoting water and sanitation improvements. After the “June 26 Directive” of 1965, barefoot doctors gradually assumed some grassroots control functions (28).

A notable characteristic of this organizational system was Party-government leadership, with the heads of leading groups at all levels being Party-government leaders of the same level rather than health department officials, reflecting its positioning as a “political task.” This arrangement ensured cross-departmental coordination capacity and resource allocation authority but also brought issues of relatively insufficient professionalism. Additionally, schistosomiasis control leading groups and Patriotic Health Campaign committees often had highly overlapping membership, achieving organic integration of the two systems (15).

## 4 Key practices in mass mobilization for schistosomiasis control

Mass mobilization was the core characteristic of the anti-schistosomiasis campaign in the 1950s-1960s, with specific practical strategies encompassing four dimensions: organizational integration, health education, snail elimination campaigns, and mass treatment.

### 4.1 Integration with the Patriotic Health Campaign

A distinctive feature of the anti-schistosomiasis campaign in the 1950s-1960s was its incorporation into the Patriotic Health Campaign framework. The Patriotic Health Campaign began in 1952, initially launched in response to allegations of biological warfare during the Korean War, and gradually developed into the basic organizational form of New China’s public health work (8). This integration enabled schistosomiasis control to achieve large-scale implementation by leveraging the organizational networks and political legitimacy already established by the Patriotic Health Campaign. After schistosomiasis control was established as a national priority in 1955, Patriotic Health Campaign committees in endemic areas incorporated it into annual work plans, utilizing existing networks to mobilize community participation in snail elimination, water and sanitation improvements, and environmental remediation (29). In practice, schistosomiasis control leading groups and Patriotic Health Campaign committees often had highly overlapping membership, achieving integration of resources and forces. Mass mobilization experience accumulated by the Patriotic Health Campaign in the early 1950s—including political mobilization, health education, competitions and evaluations, and model demonstrations—was directly transferred to schistosomiasis control work (30).

Incorporating schistosomiasis control into the Patriotic Health Campaign framework gave it political significance beyond mere disease control. Participation in the Patriotic Health Campaign was viewed as a patriotic act and civic duty, and schistosomiasis control was thus imbued with political meanings of “transforming the backward face of old China” and “building a new socialist countryside.” This politicized discourse strategy played an important role in mobilizing peasant participation in snail elimination labor (17). As a long-term institutional arrangement, the Patriotic Health Campaign committees at all levels became standing institutions, providing sustained organizational support for schistosomiasis control. However, the generalized objectives of the Patriotic Health Campaign may have diluted attention to schistosomiasis as a specific disease, making it difficult to fully meet specialized needs.

### 4.2 Health education and propaganda activities

Rural illiteracy rates in 1950s China reached approximately 80%, and peasants generally lacked understanding of schistosomiasis transmission mechanisms and prevention methods; in some areas, the disease was even attributed to superstitious beliefs (8). In this context, health education became an important component of mass mobilization, with goals extending beyond transmitting disease knowledge to changing peasant health concepts and behavioral habits. Facing populations with high illiteracy rates, health education adopted highly visual communication strategies. Propaganda posters were the most representative medium, using vibrant colors, simple images, and accessible slogans to visually demonstrate schistosomiasis transmission routes, snail identification features, prevention measures, and treatment outcomes. Themes covered schistosome life cycle education, snail elimination campaign promotion, prevention measure dissemination, and “Farewell to the God of Plague” themed political propaganda (47). Beyond propaganda posters, health education also employed multiple communication channels adapted to rural environments, including theatrical and artistic performances, film screenings, radio broadcasts, and village meetings. Local cultural propaganda teams created programs themed around schistosomiasis using storytelling narratives to transmit disease knowledge, and mobile film screening teams touring rural areas had a powerful educational effect on peasants who had never been exposed to modern medical knowledge (8).

Diversified health education strategies played a positive role in improving peasant disease awareness. According to historical literature, knowledge awareness rates regarding schistosomiasis transmission routes, snail identification, and prevention measures improved among peasants in endemic areas following systematic health education (31). However, due to the lack of systematic survey data from the 1950s-1960s, the actual coverage and knowledge transmission effectiveness of health education cannot be precisely quantified. Additionally, gaps existed between knowledge transmission and behavior change, as peasants still found it difficult to completely avoid exposure risks under the constraints of production and living conditions; some health education content carried obvious political propaganda overtones with insufficient scientific accuracy; and health education coverage and sustainability varied significantly across different areas.

### 4.3 Community participation in snail elimination

The Oncomelania hupensis snail is the only intermediate host of Schistosoma japonicum in China, and eliminating snails is a key element in interrupting the transmission chain. The 1950s-1960s campaign adopted large-scale community snail elimination as a core strategy, mobilizing millions of peasants to participate. Community snail elimination used agricultural cooperatives (and people’s communes after 1958) as the basic organizational unit. During agricultural slack seasons, production teams organized laborers for collective snail habitat clearance based on tasks assigned from above. Snail elimination campaigns had distinct militarized characteristics, with production teams organized into “snail elimination assault teams” under unified command of team leaders, conducting labor competitions to select “snail elimination models” (32). According to statistics, during the campaign peak of 1956–1958, hundreds of millions of peasant labor-days were invested in the five provinces of Hubei, Hunan, Jiangxi, Anhui, and Jiangsu alone, with annual participation reaching millions of person-times (33). Snail elimination measures primarily included physical and chemical methods. Physical methods were the main approach, including soil burial, burning, filling old habitats while creating new ones, and plowing with sun exposure. These were technically simple and low-cost, suitable for promotion under conditions of scarce professional equipment and drugs, but required substantial labor inputs and effectiveness was affected by environmental conditions. Notably, the implementation of snail elimination and treatment strategies varied substantially across the three endemic types. In hilly and waterway endemic areas, where snail habitats were relatively confined and accessible, physical methods such as soil burial and channel hardening proved highly effective, enabling several counties to achieve transmission control or elimination by the early 1960s. In contrast, lake and marshland endemic areas—particularly the Dongting Lake and Poyang Lake regions—presented far greater challenges due to vast, seasonally flooded snail-breeding habitats that were difficult to modify permanently. Mass mobilization campaigns in these areas invested enormous labor inputs in land reclamation and embankment construction, yet snail repopulation often occurred after seasonal flooding, limiting long-term effectiveness (9, 32). Similarly, mass treatment coverage and compliance varied by region: hilly areas with concentrated populations achieved higher treatment rates, while dispersed settlements in lake regions posed logistical challenges for mobile medical teams (34). Chemical methods primarily used molluscicides such as sodium pentachlorophenate and calcium cyanamide, but these presented problems of high toxicity, significant environmental impact, and relatively high costs (31).

An important innovation of the anti-schistosomiasis campaign was the organic integration of snail elimination with agricultural production and water conservancy construction. “Combining snail elimination with land reclamation” transformed snail-breeding wetlands into farmland through lake reclamation; “combining fecal management with agricultural fertilization” promoted harmless fecal treatment technologies that both interrupted transmission routes and addressed fertilizer needs; hardening of irrigation channels eliminated snail breeding conditions in earthen channels; and reservoir construction and river channel regulation changed hydrological conditions to make them unfavorable for snail breeding (35). This integration strategy linked schistosomiasis control with peasants’ immediate interests, enhancing participation motivation and reducing marginal costs. However, some lake reclamation projects, if improperly planned, could conversely expand snail distribution ranges. Additionally, the sustainability of snail elimination effectiveness was insufficient, with resurgence in some areas after the campaign momentum subsided, and extremely difficult snail elimination in lake/marshland heavily endemic areas due to complex ecological environments (32). Notably, the management of animal reservoirs—particularly the estimated 1.2 million infected water buffaloes—remained a significant gap in the campaign. While some localities implemented livestock chemotherapy and restricted grazing in snail-infested areas, these measures were neither systematic nor widely enforced (36). The mass mobilization model faced an inherent tension between agricultural production goals, which depended on maintaining water buffalo health and draft power, and schistosomiasis control objectives that required limiting bovine access to contaminated water sources. This tension reflected a broader limitation: the campaign’s emphasis on snail elimination and human treatment inadequately addressed the zoonotic nature of Schistosoma japonicum, a gap that contemporary “One Health” approaches now seek to remedy (37).

### 4.4 Mass treatment and case management

Mass population treatment was another core component of schistosomiasis control. Treating symptomatic patients not only improved individual health but also reduced transmission intensity by decreasing egg output. The main treatment drugs in the 1950s-1960s were antimony compounds, including antimony potassium tartrate (APT) and sodium stibogluconate, which had good efficacy against adult schistosomes but severe toxic side effects, commonly including nausea and vomiting, abdominal pain and diarrhea, cardiac arrhythmias, and liver and kidney damage, with severe cases potentially fatal (27). The “three-day regimen” was promoted after 1957, compressing the total antimony dose into 3 days, shortening the treatment cycle and making large-scale mobile treatment possible. The treatment landscape was not fundamentally transformed until the successful development of praziquantel in the late 1970s. Mass treatment primarily relied on the “mobile medical team” model, with county-level control stations or county hospitals organizing medical personnel to go deep into endemic villages for concentrated treatment, providing hospitalization or observation treatment for those confirmed infected through fecal examination. Mobile medical team dispatch was typically combined with campaign-style mass surveys and treatment, with counties organizing concentrated actions such as “hundred-day battles” and “winter offensives” (34).

However, mass treatment faced the challenge of peasant resistance. Fear of adverse reactions was the primary reason—severe reactions to antimony treatment were widely known in rural areas, with reports of treatment-related deaths in some areas (27). Conflicts with agricultural production were also an important factor, as treatment requiring hospitalization for several days affected agricultural labor. Additionally, some mildly symptomatic or asymptomatic infected individuals lacked awareness of their infection, resulting in low willingness to cooperate. Response strategies included ideological mobilization, model demonstrations, administrative measures, and service improvements, but the use of coercive measures also raised ethical concerns (34).

## 5 SWOT analysis of mass mobilization mechanisms

Based on the above historical analysis, this review applied the SWOT framework to systematically evaluate the strengths, weaknesses, opportunities, and threats of the mass mobilization model from both internal factors and external environment dimensions (Table 2).

**Table 2:** SWOT analysis of mass mobilization mechanisms in the anti-schistosomiasis campaign.

Strengths	Weaknesses
Extensive population coverage through existing political and administrative networks	Insufficient professional and technical expertise at grassroots level
Low-cost resource mobilization utilizing collective labor during agricultural slack seasons	Public resistance to treatment due to severe side effects of antimony compounds
Integration with agricultural production aligned economic and health interests	Campaign-style approach lacked long-term sustainability mechanisms
Strong political authority from highest leadership ensured compliance and participation	Formalism in local implementation; exaggerated reporting of achievements
Effective visual health communication adapted to populations with high illiteracy rates	Over-emphasis on snail elimination with insufficient attention to case treatment
Opportunities	Threats
National reconstruction agenda provided strong political impetus for disease control	Great Leap Forward (1958–1960) diverted resources and attention from systematic health work
Agricultural development goals aligned with schistosomiasis elimination targets	Political campaigns and mass movements interfered with professional disease control
Early successes (e.g., Yujiang County 1958) generated momentum and demonstration effect	Environmental factors (flooding, irrigation expansion) caused disease resurgence
Socialist bloc experience provided organizational models for mass mobilization	Limited availability of effective and safe drugs constrained treatment options

Analysis synthesized from historical literature.

### 5.1 Strengths

The most notable strength of the mass mobilization model lay in its coverage. Leveraging existing networks including Communist Party grassroots organizations, Patriotic Health Campaign committees, and agricultural cooperatives, schistosomiasis control information and activities could rapidly reach hundreds of millions of rural people—a coverage capacity unmatched by any professional medical system at the time (38). The mass mobilization model fully utilized the rural collectivization system and seasonal characteristics of agricultural production, organizing large-scale unpaid or low-cost labor during agricultural slack seasons. Physical methods for snail elimination had low technical thresholds and did not require expensive equipment or drugs; it is estimated that the labor-days invested in community snail elimination in the 1950s-1960s were equivalent to billions of yuan in value (33).

The integration of schistosomiasis control with agricultural production and water conservancy construction was an important innovation, making it an organic component of agricultural productivity increases and rural development. Peasant participation in snail elimination was not only a political task but could also bring direct economic benefits. Chairman Mao’s direct attention to schistosomiasis control and the directive “Schistosomiasis must be eliminated” endowed this work with the highest level of political authority, with cadres at all levels treating it as an important political task (17). Highly visual, localized communication strategies transmitted disease knowledge to rural populations with 80% illiteracy rates through propaganda posters, theatrical performances, and films, effectively raising peasant awareness levels.

### 5.2 Weaknesses

The mass mobilization model had obvious deficiencies in professional technical depth. In the 1950s, schistosomiasis control professionals were concentrated primarily at provincial and county institutions, with virtually no systematically trained professionals below the township level. Grassroots snail elimination campaigns relied mainly on village cadres and ordinary peasants, lacking in-depth understanding of snail ecology and behavior (34). Severe adverse reactions to antimony treatment were the main obstacle to mass treatment, not only affecting peasant treatment compliance but also creating negative impressions of schistosomiasis treatment.

The mass mobilization model had distinct “campaign” characteristics—concentrating forces, short-term assaults, and pursuing rapid results—which were difficult to sustain. After the campaign peak, political attention shifted, resource inputs decreased, and grassroots implementation intensity declined, with some achievements experiencing reversal (31). Under pressure to demonstrate political achievements and meet targets, problems of formalism (xingshi zhuyi)—a phenomenon in which local officials created a facade of compliance by reporting inflated statistics, conducting superficial inspection-oriented activities rather than substantive control work, and prioritizing the appearance of achievement over actual effectiveness—and exaggerated reporting emerged in some areas, particularly severe during the Great Leap Forward period. The 1963 central government review found that some counties and cities declared to have “eliminated” the disease had actually experienced resurgence, necessitating strategy adjustments emphasizing consolidation of achievements (15). Additionally, control strategies in the 1950s-1960s emphasized snail elimination as the top priority, with relatively insufficient attention to case treatment and management—a strategic bias that weakened the long-term effectiveness of snail elimination.

### 5.3 Opportunities

The 1950s were the early period following the founding of New China, with high enthusiasm for national construction. Schistosomiasis elimination was incorporated into the grand narrative of national reconstruction and modernization, linked with the political goal of “transforming the backward face of old China,” providing unprecedented political impetus for control work (29). The “National Program for Agricultural Development (1956-1967)” proposed schistosomiasis elimination alongside agricultural development goals, providing policy basis for their integration. Yujiang County’s 1958 declaration of schistosomiasis elimination, followed by Chairman Mao’s composition of “Farewell to the God of Plague,” generated a powerful demonstration effect, proving that schistosomiasis “can be eliminated” and inspiring control efforts in other endemic areas (17). The Soviet Union and other socialist countries’ experience in mass mobilization for public health provided organizational model references for China.

### 5.4 Threats

The Great Leap Forward movement beginning in 1958 had complex impacts on schistosomiasis control. The excessive emphasis on steel production and agricultural high yields during the Great Leap Forward diverted attention from health work, with some areas reassigning schistosomiasis control professionals to participate in the “mass steel production” campaign. The failure of the Great Leap Forward and the 3 years of difficulty (1959–1961) led to massive rural population deaths, with control work nearly at a standstill. Frequent political movements in the 1950s-1960s (Anti-Rightist Campaign, Great Leap Forward, Four Cleanups Campaign, Cultural Revolution, etc.) caused continuous interference with professional work, with professional technicians often becoming targets of criticism. After the outbreak of the Cultural Revolution in 1966, schistosomiasis control institutions at all levels fell into paralysis.

Schistosomiasis transmission is closely related to hydrological environment. Seasonal flooding is frequent in the middle and lower Yangtze River region, with floodwaters capable of spreading snails and schistosomes to broader areas, undoing achievements in snail elimination. Expansion of irrigation agriculture in some areas conversely expanded snail breeding environments. The lack of safe and effective anti-schistosomal drugs was a fundamental constraint on mass treatment—the severe toxicity of antimony compounds made mass treatment high-risk work, a technological constraint not overcome until the advent of praziquantel in the late 1970s.

## 6 Discussion

### 6.1 Factors potentially associated with campaign effectiveness

**Figure 2:**
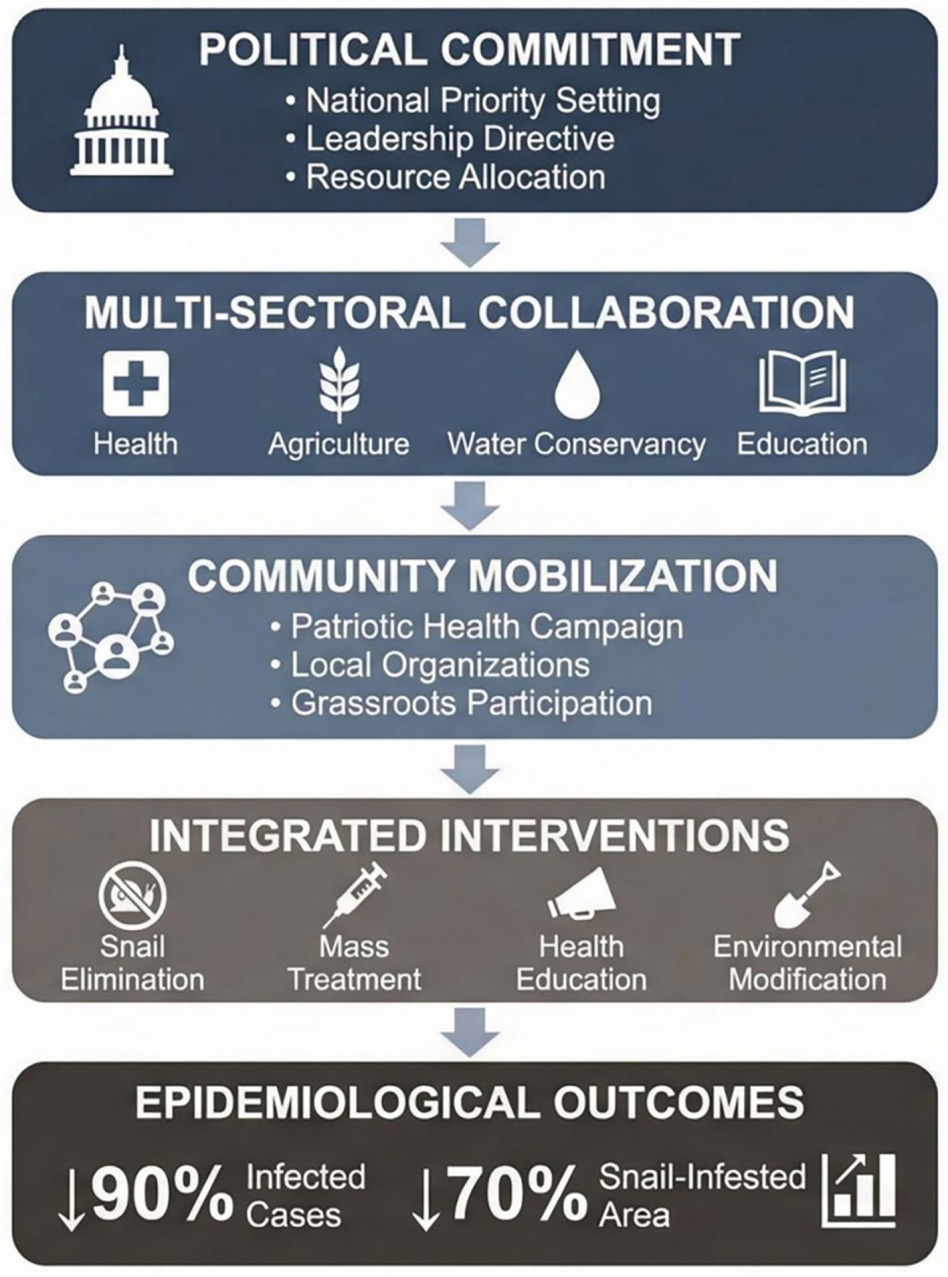
Logical model: factors potentially associated with the effectiveness of mass mobilization in schistosomiasis control.

Through systematic review of China’s anti-schistosomiasis campaign in the 1950s-1960s, this study identified several factors potentially associated with the effectiveness of the mass mobilization model. It should be noted that as this review employed a scoping review methodology based on synthesis of historical literature, the relationships between these factors and epidemiological outcomes are primarily based on historical narrative and logical inference rather than rigorous causal inference. These factors appear to be interrelated and may form a multi-level logical pathway from political commitment to epidemiological outcomes (Figure 2). It should be emphasized that the relationships depicted represent plausible associations based on historical narrative rather than empirically verified causal pathways.

High-level political commitment may have served as a core driver of the entire control system. Chairman Mao’s directive “Schistosomiasis must be eliminated” elevated this disease control work to national strategic importance, with Party-government leaders at all levels treating it as an important political task, ensuring the effectiveness of resource allocation and organizational mobilization (9). This experience is consistent with findings from contemporary global health governance research suggesting that high-level political commitment may be an important prerequisite for successful NTD elimination programs (39). Multi-sectoral coordination mechanisms appear to have enabled schistosomiasis control to transcend the functional scope of the health department alone, coordinating health, agriculture, water conservancy, education, and other departments through the central schistosomiasis control leading group to form a joint force. This cross-departmental integration model is highly compatible with contemporary “One Health” concepts and “Health in All Policies” advocacy (40). Linking disease control goals with agricultural productivity increases and rural development was another key innovation. Strategies such as combining snail elimination with land reclamation and fecal management with agricultural fertilization enabled peasant participation in control to yield not only political obligations but also direct economic benefits, which may have contributed to reducing mobilization costs and enhancing participation enthusiasm. Facing the reality of 80% rural illiteracy, highly visual, localized health communication strategies transmitted disease knowledge through forms readily accepted by peasants—including propaganda posters, theatrical performances, and films—embodying the principle of cultural appropriateness in health communication.

### 6.2 Challenges and lessons from the campaign

The SWOT analysis in this review reveals the inherent tensions and historical limitations faced by the mass mobilization model, providing important references for understanding the applicable boundaries of campaign-style public health interventions.

The tension between mass participation and scientific professionalism was a core contradiction throughout the control campaign. Large-scale mass mobilization achieved population coverage that professional medical systems could not match, but grassroots implementers generally lacked professional understanding of snail ecology and disease transmission patterns, resulting in some snail elimination campaigns being inefficient or even counterproductive. This issue remains universally relevant in contemporary community-participatory public health interventions—how to ensure technical quality of interventions while expanding participation scope is an issue requiring ongoing attention in community mobilization program design. The sustainability deficits of the campaign-style governance model also warrant deep reflection. This review found that schistosomiasis control made significant progress during the campaign peak of 1956–1958, but after the campaign momentum subsided, political attention shifted, resource inputs decreased, and disease resurgence occurred in some areas. The 1963 central government review found that counties and cities previously declared to have “eliminated” the disease had actually experienced resurgence, necessitating strategy adjustments emphasizing consolidation of achievements. This historical lesson demonstrates that while “campaign-style” interventions can concentrate resources to achieve breakthroughs in the short term, lack of institutionalized, normalized maintenance mechanisms makes it difficult to consolidate achievements. Global malaria elimination and lymphatic filariasis elimination programs face similar challenges when entering the “last mile” phase (41). The formalism and exaggerated reporting problems that emerged in some areas under political pressure damaged the accuracy of surveillance data, serving as a warning for contemporary public health surveillance system design—there is a need to establish professional disease surveillance systems independent of administrative performance evaluation. The SWOT analysis also revealed two additional issues worthy of attention. Control strategies in the 1950s-1960s over-emphasized snail elimination while giving insufficient attention to case treatment—a strategic bias partly stemming from peasant resistance due to severe adverse reactions to antimony treatment, and also reflecting the policy orientation of the “prevention-first” health policy at the time. The hydrological environment characteristics of the middle and lower Yangtze River region—seasonal flooding and irrigation agriculture expansion—posed continuous threats to the sustainability of snail elimination achievements, suggesting that disease control strategies need to fully consider the dynamic changes of ecological environments.

### 6.3 Implications for contemporary global public health

Although China’s anti-schistosomiasis campaign of the 1950s-1960s occurred under specific political and economic conditions, its historical experience retains multifaceted relevance for contemporary global public health practice (Table 3).

**Table 3:** Lessons from 1950s-1960s China for contemporary global public health.

Historical experience	Contemporary application	Target context
High-level political commitment and national priority setting	Advocacy for government ownership and sustained leadership	NTD elimination programs in endemic countries
Multi-sectoral integration (health, agriculture, water)	One Health approach; Health in All Policies	Zoonotic disease control; environmental health
Mass community mobilization and participation	Community-based surveillance and participatory approaches	Ebola, COVID-19 response; vaccine campaigns
Health education adapted to local literacy levels	Culturally appropriate communication strategies	Low-resource settings with limited health infrastructure
Integration of health goals with economic development	Health-development co-benefits framing	SDG implementation; addressing poverty-disease nexus
Limitations of campaign-style approach	Need for institutionalized, sustainable health systems	Post-elimination surveillance; health system strengthening
Animal reservoir management gap	Integrated human-animal surveillance; One Health implementation	Zoonotic NTD programs; livestock-endemic regions

Implications derived from historical analysis and contemporary global health literature.

In the field of NTD elimination, the importance of high-level political commitment and national priority agenda-setting is widely recognized. The WHO 2021–2030 Roadmap for Neglected Tropical Diseases explicitly identifies political commitment as a key element for achieving elimination targets, and China’s historical experience in schistosomiasis control provides a replicable mobilization model for resource-limited countries (WHO, 2021). Recent WHO progress reports confirm that sustained commitment and integrated approaches remain central to advancing global schistosomiasis elimination (42). The experience of multi-sectoral collaboration has direct reference value for contemporary “One Health” practice. As a zoonotic parasitic disease, effective schistosomiasis control itself requires coordination among health, agriculture, water conservancy, environmental, and other sectors. While the cross-departmental coordination mechanisms established in 1950s-1960s China carried characteristics of the planned economy era, its core concept of integrating resources and interests across different departments remains applicable in contemporary zoonotic disease control and environmental health governance (37). Community mobilization and participatory approaches have been widely applied in recent global health emergency responses. Both the 2014–2016 West African Ebola outbreak and the COVID-19 pandemic since 2019 have demonstrated that top-down interventions lacking community trust and participation have difficulty achieving expected results. China’s anti-schistosomiasis campaign experience of changing awareness through health education, enhancing motivation through interest alignment, and achieving coverage through organizational networks provides historical reference for contemporary community-participatory interventions (43).

Notably, contemporary Chinese rural community health education programs continue the core experiences of the 1950s-60s. Li et al. (44) studied community health education programs in impoverished counties in southwestern China, demonstrating that health education teams composed of village-level leaders and grassroots health workers transmitted health information through lectures, brochures, and training. Health literacy in the intervention group significantly increased from 1.92% in 2019 to 17.71% in 2021, confirming the continued effectiveness of localized community health education strategies in resource-poor areas (44).

Political mobilization models continue to play an important role in contemporary Chinese public health emergency responses. Gao and Zhang (45) analyzed China’s public health policies during the COVID-19 pandemic, demonstrating that the government’s powerful social mobilization capacity in crisis management has historical continuity with the 1950s-60s anti-schistosomiasis campaign, while also facing new challenges in the digital governance era, including balancing individual freedom with public health and the equitable application of digital technologies (45).

At the same time, this review also highlights boundary conditions for applying historical experience. The limitations of campaign-style interventions indicate that public health system construction needs to transition from “campaign-style” to “institutionalized,” establishing sustainable human resources, fiscal investment, and monitoring and evaluation mechanisms. Indeed, China’s own post-1980s trajectory illustrates this transition: following the end of the mass campaign era, schistosomiasis control gradually shifted toward institutionalized approaches characterized by the establishment of permanent professional control stations, routine surveillance systems, integration of praziquantel-based chemotherapy into primary healthcare, and evidence-based stratified control strategies adapted to different endemic settings (12, 28). This evolution from mobilization-driven campaigns to science-based institutional management represents a critical policy transition that enabled sustained progress toward elimination. The health system strengthening and universal health coverage emphasized in contemporary global health agendas are precisely responses to this historical lesson (46). Additionally, China’s political system and social mobilization capacity in the 1950s-1960s had historical specificity, and the transferability of this experience to other countries and regions requires careful assessment—under democratic governance frameworks, community mobilization needs to be based more on principles of voluntary participation and benefit-sharing rather than administrative orders and political pressure.

### 6.4 Limitations

This study has several limitations that should be acknowledged. As a scoping review primarily relying on secondary literature, access to original archival data from the 1950s-1960s was not possible, and the accuracy of historical data is constrained by the quality of source documents. Given the political environment of that era, some epidemiological data may have been subject to exaggeration or distortion; this review therefore maintains a cautious approach when citing such data. While this study focuses on macro-level analysis of mass mobilization mechanisms, it does not fully explore the differential experiences across different endemic types (lake/marshland, hilly, and waterway regions). The methodological positioning of scoping review limits the rigor of causal inference; the associations identified in this review between various factors and epidemiological outcomes require further verification through more rigorous research designs. Due to language limitations, this review may have missed some important historical documents published only in Chinese. Although this review includes a discussion of animal reservoir management strategies, the analysis remains limited due to the scarcity of systematic historical data on livestock interventions during the 1950s–1960s campaign period, which warrants further investigation in future studies.

## 7 Conclusion

This review systematically examined the development trajectory, mass mobilization practices, and effectiveness and limitations of China’s anti-schistosomiasis campaign in the 1950s-1960s using a scoping review methodology. The findings indicate that under conditions of scarce medical resources and rural illiteracy rates reaching 80%, China successfully mobilized millions of peasants to participate in large-scale snail elimination and treatment campaigns through high-level political commitment, multi-sectoral coordination mechanisms, integration of disease control with economic development, and localized health communication strategies, achieving remarkable epidemiological outcomes with the number of infected people declining from 11.6 million to below 1 million by approximately 1982–1983 and snail-infested areas decreasing by over 75%. This historical experience demonstrates that political mobilization and community participation can partially compensate for the shortage of professional medical resources, providing a feasible pathway for disease control in resource-limited countries. However, the SWOT analysis in this review also revealed inherent limitations of the mass mobilization model—weak professional and technical capacity at the grassroots level, lack of sustainable mechanisms in the campaign-style approach, and formalism and exaggerated reporting that compromised the accuracy of surveillance data, leading to disease resurgence in some areas after the campaign momentum subsided. This historical lesson suggests that effective public health interventions require a balance between broad community participation and scientific professionalism, as well as the establishment of institutionalized, normalized maintenance mechanisms. In the context of contemporary global health governance, the historical experience identified in this review retains reference value for NTD elimination programs, “One Health” practice, and public health emergency response, though its application requires careful consideration of contextual differences in political systems and social mobilization capacity. Future research could further compare different pathways of schistosomiasis control between China and other countries, conducting in-depth analysis of the specific mechanisms and conditions for institutional transition.

## Data Availability

The original contributions presented in the study are included in the article/supplementary material, further inquiries can be directed to the corresponding author/s.

## References

[1] World Health Organization. Global Report on Neglected tropical Diseases 2024. Geneva: WHO (2024).

[2] Xu J Steinmann P Maybe D Zhou XN Lv S Li SZ . Evolution of the national schistosomiasis control programmes in China. Adv Parasitol. (2016) 92:1–38. doi: 10.1016/bs.apar.2016.02.001, 27137441

[3] Zhang LJ Xu ZM Qian YJ Dang H Lu S Xu J . Endemic status of schistosomiasis in people’s republic of China in 2021. Chin J Schisto Control. (2022) 34:1–8.

[4] Collins C Xu J Tang S. Schistosomiasis control and the health system in P.R. China. Infect Dis Poverty. (2012) 1:8. doi: 10.1186/2049-9957-1-8, 23849320 PMC3710143

[5] Katz N. Schistosomiasis control in Brazil. Mem Inst Oswaldo Cruz. (1998) 93:33–5. doi: 10.1590/S0074-02761998000700005 9921321

[6] Olveda RM Acosta LP Tallo V Baltazar PI Lesiguez JL Estanislao GG . Bilharzia in the Philippines: past, present, and future. Int J Infect Dis. (2014) 18:52–6. doi: 10.1016/j.ijid.2013.09.011, 24211228

[7] Barakat RMR. Epidemiology of schistosomiasis in Egypt: travel through time. J Adv Res. (2013) 4:425–32. doi: 10.1016/j.jare.2012.07.003, 25685449 PMC4293883

[8] Gross M. Farewell to the God of Plague: Chairman Mao’s Campaign to Deworm China. Oakland: University of California Press (2016).

[9] Xu J Li SZ Guo JG Zhou XN Garba Djirmay A. The WHO new guideline to control and eliminate human schistosomiasis: implications for the verification of transmission interruption and surveillance of *Schistosoma japonicum* in China. Infect Dis Poverty. (2022) 11:79. doi: 10.1186/s40249-022-01003-w, 35778748 PMC9247933

[10] Chen J Xu J Bergquist R Li SZ Zhou XN. Farewell to the god of plague: the importance of political commitment towards the elimination of schistosomiasis. Trop Med Infect Dis. (2018) 3:108. doi: 10.3390/tropicalmed3040108, 30282897 PMC6306784

[11] Cao CL Zhang LJ Deng WP Li YL Lv C Dai SM . Contributions and achievements on schistosomiasis control and elimination in China by NIPD-CTDR. Adv Parasitol. (2020) 110:1–62. doi: 10.1016/bs.apar.2020.04.002, 32563322

[12] Xu J Lv S Cao CL Li SZ Zhou XN. Elimination of schistosomiasis in China: current status and future prospects. PLoS Negl Trop Dis. (2021) 15:e0009578. doi: 10.1371/journal.pntd.0009578 34351907 PMC8341657

[13] Luo F Yang W Yin M Hu W Lin D Zhou X . A new era of schistosomiasis control and elimination in China: challenges and strategies. Lancet Reg Health West Pac. (2024) 45:101068. doi: 10.1016/j.lanwpc.2024.101068

[14] World Health Organization. Ending the Neglect to Attain the Sustainable Development Goals: a Road Map for Neglected Tropical Diseases 2021–2030. Geneva: WHO (2021).

[15] Liang S Yang CH Zhong B Guo JG Li HZ Carlton EJ . Surveillance systems for neglected tropical diseases: global lessons from China’s evolving schistosomiasis reporting systems, 1949-2014. Emerg Themes Epidemiol. (2021) 11:15. doi: 10.1186/1742-7622-11-19 PMC4531518 26265928

[16] Zhang LJ He JY Yang F Dang H Li YL Guo SY . Progress of schistosomiasis control in People's Republic of China in 2022. Chin J Schistosomiasis Control. (2023) 35:217–24. doi: 10.16250/j.32.1374.2023073, 37455091

[17] Fan K Lai HK. Mao Zedong’s fight against schistosomiasis. Perspect Biol Med. (2008) 51:176–87. doi: 10.1353/pbm.0.0013, 18453723

[18] Zhou L. The cultural policies of schistosomiasis control in China: a historical analysis. Parasitol Res. (2023) 122:2457–65. doi: 10.1007/s00436-023-07966-5, 37676304

[19] Ding X. Between politics and prevention: a re-examination of China’s schistosomiasis control campaign in the 1950s. Endeavour. (2019) 43:100696. doi: 10.1016/j.endeavour.2019.100696 31735367

[20] Tricco AC Lillie E Zarin W O’Brien KK Colquhoun H Levac D . PRISMA extension for scoping reviews (PRISMA-ScR): checklist and explanation. Ann Intern Med. (2018) 169:467–73. doi: 10.7326/M18-0850 30178033

[21] Peters MDJ Godfrey C McInerney P Munn Z Tricco AC Khalil H. Best practice guidance and reporting items for the development of scoping review protocols. JBI Evid Synth. (2020) 18:2119–26. doi: 10.11124/JBIES-20-00167 35102103

[22] Popay J Roberts H Sowden A Petticrew M Arai L Rodgers M . Guidance on the conduct of Narrative Synthesis in systematic Reviews. Lancaster, UK: ESRC Methods Programme (2006).

[23] Gurel E Tat M. SWOT analysis: a theoretical review. J Int Soc Res. (2017) 10:994–1006. doi: 10.17719/jisr.2017.1832

[24] Tao B Jiang QL Luo CJ Yin ZH Wang JJ. Endemic status of schistosomiasis in lake and marshland regions of China. Acta Trop. (2021) 214:105754. doi: 10.1016/j.actatropica.2020.105754

[25] Li FY Hou XY Tan HZ Williams GM Gray DJ Gordon CA . Current status of schistosomiasis control and prospects for elimination in the Dongting Lake region of the people’s republic of China. Front Immunol. (2020) 11:574136. doi: 10.3389/fimmu.2020.574136, 33162989 PMC7583462

[26] Xu J Li SZ Zhang LJ Bergquist R Dang H Wang Q . Surveillance-based evidence: elimination of schistosomiasis as a public health problem in the people’s republic of China. Infect Dis Poverty. (2020) 9:63. doi: 10.1186/s40249-020-00676-5, 32505216 PMC7275476

[27] Ross AGP Bartley PB Sleigh AC Olds GR Li YS Williams GM . Schistosomiasis. N Engl J Med. (2002) 346:1212–20. doi: 10.1056/nejmra012396, 11961151

[28] Sun LP Xu J Cao CL Li SZ Liang YS. Approaches being used in the national schistosomiasis elimination programme in China: a review. Infect Dis Poverty. (2022) 11:45. doi: 10.1186/s40249-017-0271-9 28292327 PMC5351197

[29] Hipgrave D. Communicable disease control in China: from Mao to now. J Glob Health. (2011) 1:224–38. doi: 10.1056/NEJMhpr051833, 23198121 PMC3484775

[30] Rogaski R. Hygienic Modernity: Meanings of Health and Disease in Treaty-Port China. Berkeley: University of California Press (2004).

[31] Utzinger J Zhou XN Chen MG Bergquist R. Conquering schistosomiasis in China: the long march. Acta Trop. (2005) 96:69–96. doi: 10.1016/j.actatropica.2005.08.004, 16312039

[32] Zhou XN Wang LY Chen MG Wu XH Wang TP Lin DD . The public health significance and control of schistosomiasis in China-then and now. Acta Trop. (2005) 96:97–105. doi: 10.1016/j.actatropica.2005.07.005, 16125655

[33] Liu R Dong HF Jiang MS. The new national integrated strategy for schistosomiasis control and research progress. Chin J Schisto Control. (2016) 28:229–32.

[34] Chen MG Feng Z. Schistosomiasis control in China. Parasitol Int. (1999) 48:11–9. doi: 10.1016/S1383-5769(99)00004-5 11269321

[35] Wang LD Guo JG Wu XH Chen HG Wang TP Zhu SP . China’s new strategy to block Schistosoma japonicum transmission: experiences and impact beyond schistosomiasis. Trop Med Int Health. (2009) 14:1475–83. doi: 10.1111/j.1365-3156.2009.02403.x, 19793080

[36] Gray DJ Williams GM Li YS McManus DP. Transmission dynamics of Schistosoma japonicum in the lakes and marshlands of China. PLoS One. (2008) 3:e4058. doi: 10.1371/journal.pone.0004058, 19115007 PMC2605259

[37] Hong Z Li L Zhong LJ Wang Q Xu J Li SZ . Elimination of schistosomiasis japonica in China: from the One Health perspective. China CDC Wkly. (2022) 4:130–4. doi: 10.46234/ccdcw2022.024 35265392 PMC8886488

[38] Jamison DT Evans JR King T Porter I Prescott N Prost A. China: the Health Sector. Washington: World Bank (1984).

[39] Engels D Zhou XN. Neglected tropical diseases: an effective global response to local poverty-related disease priorities. Infect Dis Poverty. (2020) 9:10. doi: 10.1186/s40249-020-0630-9, 31987053 PMC6986060

[40] Zinsstag J Schelling E Crump L Waltner-Toews D Tanner M. One Health: the Theory and Practice of Integrated Health Approaches. 2nd ed. Wallingford: CABI (2023).

[41] Rollinson D Knopp S Levitz S Stothard JR Tchuem Tchuente LA Garba A . Time to set the agenda for schistosomiasis elimination. Acta Trop. (2022) 228:106316. doi: 10.1016/j.actatropica.2021.106316 22580511

[42] WHO. Schistosomiasis: progress report 2001-2011, strategic plan 2012-2020. Wkly Epidemiol Rec. (2025) 100:35–50.

[43] Bardosh KL de Vries DH Kaberry J Liang L Sighoko D Okwi A . Rethinking NTD programme design: implications for global health through the lens of community participation. PLoS Negl Trop Dis. (2020) 14:e0007983. doi: 10.1371/journal.pntd.0007983 32106219 PMC7046186

[44] Li Y Huang K Ling Y Jiao F Fu H Deng J. The effect of community-based health education programs on health literacy in severely impoverished counties in southwestern China: results from a quasi-experimental design. Front Public Health. (2023) 10:1057386. doi: 10.3389/fpubh.2022.1088934 PMC9871388 36703836

[45] Gao X Zhang Y. China’s public health policies in response to COVID-19: from an “authoritarian” perspective. Front Public Health. (2021) 9:756677. doi: 10.3389/fpubh.2021.756677, 34976920 PMC8714736

[46] Kruk ME Gage AD Arsenault C Jordan K Leslie HH Roder-DeWan S . High-quality health systems in the sustainable development goals era: time for a revolution. Lancet Glob Health. (2018) 6:e1196–252. doi: 10.1016/S2214-109X(18)30386-3, 30196093 PMC7734391

[47] U.S. National Library of Medicine. Chinese Public Health Posters Digital Collection. Available online at: https://www.nlm.nih.gov/hmd/chineseposters/ (Accessed 20 March 2024)

